# PDIA2 Bridges Endoplasmic Reticulum Stress and Metabolic Reprogramming During Malignant Transformation of Chronic Colitis

**DOI:** 10.3389/fonc.2022.836087

**Published:** 2022-07-04

**Authors:** Jie Tao, Lin Yin, Ao Wu, Jiaoli Zhang, Jingpu Zhang, Huichun Shi, Siyuan Liu, Liangfei Niu, Li Xu, Yanling Feng, Shixian Lian, Lei Li, Liyan Zeng, Xianmin Meng, Xiaohui Zhou, Tiefu Liu, Lijun Zhang

**Affiliations:** ^1^ Scientific Research Institute, Shanghai Public Health Clinical Center, Fudan University, Shanghai, China; ^2^ The College of Information, Mechanical and Electrical Engineering, Shanghai Normal University, Shanghai, China; ^3^ Clinical Pathology Laboratory, Shanghai Public Health Clinical Center, Fudan University, Shanghai, China; ^4^ Department of Surgery, Shanghai Public Health Clinical Center, Fudan University, Shanghai, China; ^5^ Department of Clinical Research Center, Shanghai Public Health Clinical Center, Fudan University, Shanghai, China; ^6^ Animal Research Center, Shanghai Public Health Clinical Center, Fudan University, Shanghai, China

**Keywords:** chronic colitis, colon cancer, endoplasmic reticulum stress, metabolic reprogramming, malignant transformation, PDIA2

## Abstract

**Background:**

Chronic inflammation contributes to approximately 20% of cancers; the underlying mechanisms are still elusive. Here, using an animal model of colitis to colon-cancerous transformation, we demonstrated that endoplasmic reticulum (ER) stress couples with metabolic reprogramming to promote a malignant transformation of chronic inflammation.

**Methods:**

The animal model for chronic colitis to colon-cancerous transformation was established in C57BL/6N mice by azoxymethane (AOM) and dextran sodium sulfate (DSS) treatments. The differential proteins in control and AOM/DSS-treated colon mucosa were determined using proteomic analysis; the kinetics of metabolic modifications were monitored by mitochondrial oxygen flux, extracellular acidification, and targeted metabolomics; the molecule linker between ER stress and metabolic modifications were identified by coimmunoprecipitation, KEGG pathway analysis, and the subcutaneous tumor model using gene-specific knockdown colon cancer cells. Tissue array analysis were used to evaluate the differential protein in cancer and cancer-adjacent tissues.

**Results:**

AOM/DSS treatment induced 38 tumors in 10 mice at the 14th week with the mean tumor size 9.35 ± 3.87 mm^2^, which was significantly decreased to 5.85 ± 0.95 mm^2^ by the ER stress inhibitor 4-phenylbutyric acid (4PBA). Seven differential proteins were determined from control (1,067 ± 48) and AOM/DSS-treated mucosa (1,077 ± 59); the level of ER protein PDIA2 (protein disulfide isomerase-associated 2) was increased over 7-fold in response to AOM/DSS treatment. PDIA2 interacted with 420 proteins that were involved in 8 signaling pathways, in particular with 53 proteins in metabolic pathways. PDIA2 translocated from ER to mitochondria and interacted with the components of complexes I and II to inhibit oxophosphorylation but increase glycolysis. Knockdown PDIA2 in colon cancer cells restored the metabolic imbalance and significantly repressed tumor growth in the xenograft animal model. 4PBA therapy inhibited the AOM/DSS-mediated overexpression of PDIA2 and metabolic modifications and suppressed colon cancer growth. In clinic, PDIA2 was overexpressed in colon cancer tissues rather than cancer-adjacent tissues and was related with the late stages and lymph node metastasis of colon cancer.

**Conclusions:**

Persistent ER stress reprograms the metabolism to promote the malignant transformation of chronic colitis; PDIA2 serves as a molecule linker between ER stress and metabolic reprogramming. The inhibition of ER stress restores metabolic homeostasis and attenuates the cancerous transformation of chronic inflammation.

## Introduction

Cancer has long been the worldwide cause of death secondary to ischemic heart disease before age 70 and is estimated to become the first in 2060 (1). Among all types of cancers, colorectal carcinoma (CRC) ranks the third high-incidence cancer and is a leading cause of cancer-related death in older adults. The standardized incidence of CRC in 2018 in China was 23.7/100,000, placing it second in China and accounting for 28.2% of the total number of cases and 28.1% of the total number of deaths worldwide that ranks first in the world due to the large population base (2). With the development of the socioeconomic status and the aggravating trend of aging population in recent years, the incidence and mortality of CRC continue to rise, which make cancer a worldwide public health challenge.

Cancer can be induced by a number of physical, chemical, and/or biological agents, of which the inflammatory etiology has been well established in clinic (3, 4). For instance, gastric carcinoma develops from chronic gastritis by *Helicobacter pylori* infection, hepatocellular carcinoma from chronic hepatitis by hepatitis B virus infection, cervicocarcinoma from chronic cervicitis by human papilloma virus infection, and so on, in addition to colon cancer from inflammatory bowel diseases. In general, chronic inflammatory diseases contribute up to 20% of all cancers (4). Indeed, inflammation and cancers share key natures of self-sufficient growth signals, insensitivity to antigrowth signals, active proinflammatory signals, the evasion of cell apoptosis, and persistent angiogenesis (5, 6). Such a prominent link suggests close connections between inflammation and cancer.

The knowledge about how inflammation processes into cancer is incomplete so far. The inflammatory process is basically a physiological response to immunometabolic stresses that aims to eliminate inflammatory stimuli and mediators and eventually resolve inflammation and restore physiological homeostasis (7). Inflammatory signals firstly trigger a nuclear stress response and tremendously increase gene transcription. The robust increase in mRNA transcription subsequently stresses the endoplasmic reticulum (ER) to vastly process the translation, folding, sorting, and secretion of proteins and the degradation of unfolded proteins. The stress responses of nucleus and ER collaboratively form a proinflammatory “cytokine storm.” The gene transcription and protein translation are anabolic processes that need energy support; thus, mitochondrial stress rationally ensues parallel to nuclear and ER stresses to upsurge energetics to meet the energy demand of a “cytokine storm” (8). A successful resolution of inflammation restores the homeostasis of nucleus, ER, and mitochondria. However, the triad stress responses of nucleus, ER, and mitochondria also induce oxidative damage, DNA mutations, angiogenesis, and metabolic changes, if sustained in chronic inflammation, conducive to cell malignant transformation and cancer growth (9–11). The inhibition of ER stress by chemical compounds has demonstrated effective in the preclinical and clinical trials of multiple tumors (12, 13).

The ER is a network of tubules and flattened sacs that function in the quality control of cellular client proteins. When increasing in the burdens of protein translation and misfolded proteins under pathophysiological conditions, such as inflammation, ER stress, also called unfolded protein response (UPR), is triggered to restore proteostasis. A typical nature of ER stress is to overexpress protein disulfide isomerase (PDI) family members, a group of highly abundant ER enzymes that act as a chaperone, the binding partner of other proteins, disulfide isomerase in the formation of disulfide bonds, and a redox sensor (14). Chronic cytokine storm response disturbs proteostatic mechanisms that are potentially cancerogenic (15) and persistently activates and overexpresses PDI members, which have been associated with numerous human disorders, particularly in neurodegenerative diseases and cancers (14, 16). For example, the upregulated expression of PDIA1 (also known as PDI) and PDIA3 (ERp57) have been reported in the cancers of brain, lymphoma, kidney, ovarian, prostate, lung, and colon and have been considered as diagnostic biomarkers and therapeutic targets for cancer treatment and chemoprevention (17, 18). Accordingly, several attempts have been made to develop PDI inhibitors as anticancer drugs (14, 19). In addition, other UPR-associated proteins, such as glucose-regulated protein 78 (GRP 78) and molecular chaperone heat shock protein (HSP) GRP94, and ER stress signaling proteins, such as the phosphorylation of inositol-requiring transmembrane kinase/endoribonuclease 1α (IRE1α), have been reported to be upregulated in various cancers and participated in the regulation of glycolysis, the microenvironment, and immune evasion of cancers (16, 20, 21).

Inflammatory ER stress usually informs mitochondria to reprogram energetics through different pathways such as the Ca^2+^ signal transmission from ER to mitochondria *via* the mitochondria-associated membrane (MAM) and the ER protein sensors (IRE1, PERK, and ATF6), which initiate a complex transcriptional cascade (22–24). The energetics reprogramming by ER stress usually interferes mitochondrial respiration to minimize adenosine-triphosphate (ATP) production, increase reactive oxygen species (ROS) production, and switch mitochondrial energetics to glycolysis, also known as the “Warburg effect” if it occurs under normoxia that favors cell transformation and cancer development (25).

In this report, we have identified ER protein PDIA2 (also called as PDIP or PDA2) for the first time as a new contributor of chronic bowel inflammation to cancerous transformation in an animal model of colon cancer. Colon inflammation stresses ER and stimulates the overexpression of PDIA2, which translocates into mitochondria and interacts with complex components of electron transport chain, leading to the inhibition of mitochondrial respiration and energetics switch to hyperglycolysis, thereby promoting malignant transformation. The downexpression of PDIA2 restored energetic homeostasis and inhibited tumor growth. Our study highlighted the metabolic role in promoting the malignant transformation of chronic colitis.

## Materials and Methods

### Cells and Reagents

HT29 colon cancer cells and CCD-18Co normal human colon cells were obtained from American Type Culture Collection (ATCC, Shanghai, China). Colon-specific carcinogens dextran sodium sulfate (DSS; molecular weight = 36,000–50,000) and azoxymethane (AOM) (26, 27) were purchased from ICN Biomedicals Inc. (Aurora, OH, USA) and Sigma-Aldrich Company (Steinheim, Germany), respectively. The reagents for proteomics were from General Electric Company (GE) (Fort Myers, FL, USA). The primary anti-PDIA2 antibodies for Western blot and Co-IP were from Abcam company (ab223520, Cambridge, UK) and LifeSpan BioSciences, Inc (LS-C2830, Seattle, WA, USA), respectively. The β-actin antibody was from Sigma-Aldrich Company (Steinheim, Germany), Glyceraldehyde-3-phosphate dehydrogenase (GAPDH) antibody from TransGen Biotech (HC301, Beijing, China), IRE1α (14C10) Rabbit mAb from Santa Cruz (#3294, CA, USA), IRE1 alpha (phosphor Ser724) from Gen Tex (GTX132808, CA, USA), Total oxidative phosphorylation (OXPHOS) Rodent WB Antibody Cocktail from Abcam (ab110413, Cambridge, UK), Goat anti-Mouse IgG (H+L) Highly Cross-Adsorbed Secondary Antibody [Alexa Fluor 594 (A-11032)], Goat anti-Rabbit IgG (H+L) Cross-Adsorbed Secondary Antibody [Alexa Fluor 488 (A11008)] and Mito-Tracker probe (M22425) from Invitrogen company (CA, USA). 4-phenylbutyric acid (4-PBA) was from MedChemExpress Co. Ltd (Shanghai, China), transgen (AQ141) qPCR kit, peroxidase-labeled anti-rabbit IgG from Vector Laboratories (Burlingame, CA, USA), DAPI from Cell Signaling Technology (CST) (4083S, USA), and Annexin-FITC/PI from Shanghai Yisheng Biotechnology Co., Ltd (Shanghai, China). D (+)-glucose was from Wockhardt Ltd. (Maharashtra, India), and U-13C6–glucose (CLM-1396-1) from Cambridge Isotope Laboratories, Inc. (MA, USA). Succinic acid, lactic acid, aconitic acid, and fumaric acid were from MTAR Research Chemicals Inc (NJ, USA). L-malic acid was from Beijing Beina Chuanglian Biotechnology Research Institute (Beijing, China). D-fructose-6-phosphate disodium salt, 1,3-diphosphoglycerate, a-ketoglutaric acid, oxaloacetic acid, and phospho (enol) pyruvic acid cyclohexylammonium salt were from Beijing Bailingwei Technology Co., Ltd (Beijing, China). Pyruvate was from Sigma-Aldrich (SHBG2643V, USA), trisodium isocitric acid from Toronto Research Chemicals INC (Toronto, ON, Canada), and 6-phosphogluconic acid trisodium salt from Aladdin Industrial Corporation (Shanghai, China). Other analytical reagents were from Sinopharm Chemical Reagent Co., Ltd (Shanghai, China).

### Clinical Sample Collection

Ninety-six pairs of surgical colon cancer tissues and their adjacent tissues were collected for a pathological exam; the left tissues from 90 pairs were used for a microarray and 6 pairs for Western blot analysis and real-time PCR analysis according to a protocol approved by the Ethics Committee of the Shanghai Public Health Clinical Center (No. 2019-S035-02). Informed consent was obtained from all participants. All cancers were confirmed by a pathologic examination and were clinically TNM (T: primary tumor; N: regional lymph nodes; M: distant metastasis) classified according to the standard of the 7th American Joint Committee on Cancer/International Union against cancer classification (28) (**Supplement Table S1**). There were 4, 17, 44, 21, and 8 individuals who were classified as T1, T2, T3, T4a, and T4b; 50, 13, 16, 10, and 7 as N0, N1a, N1b, N2a, and N1b; and 79, 8, and 9 as M0, M1a, and M1b, respectively. A total of 38 women and 59 men with a mean age of 63.5 (26–83) years were included. Tissues without TNM information and from patients with age less than 18, pregnancy and breastfeeding, HIV, and TB infection were excluded.

### Animal Model for Malignant Transformation of Bowel Inflammation

**Figure 1:**
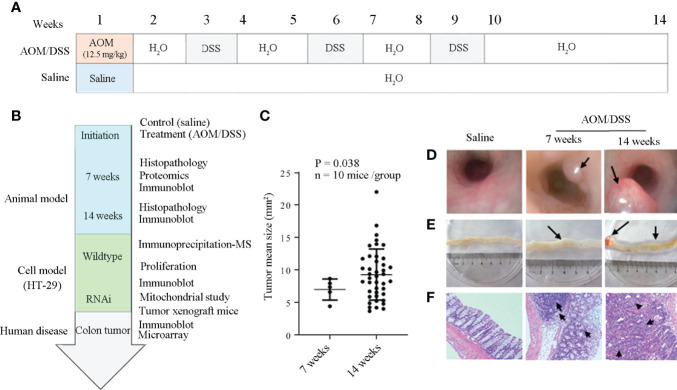
Establishment of the animal model for the malignant transformation of chronic colitis. **(A)** and **(B)** Flow chart of experiment processes. AOM, DSS, and 4PBA represent azoxymethane, dextran sodium sulfate, and 4-phenylbutyric acid, respectively. **(C)** Colon cancer development at the 7th and the 14th weeks after AOM/DSS treatment. **(D)** Colon cancer detection by colon capsule endoscopy; tumors were specified by arrows. **(E)** Anatomical observation of colon cancer; the tumors and their associated bleeding were indicated by arrows. **(F)** Histology of colon tissues. Irregular submucosa arrangement and immune cell infiltration at the 7th week, and the cells with deep-stained large nucleus and adenocarcinoma of glandular epithelium at the 14th week after AOM/DSS treatment were specified by arrows.

**Figure 2:**
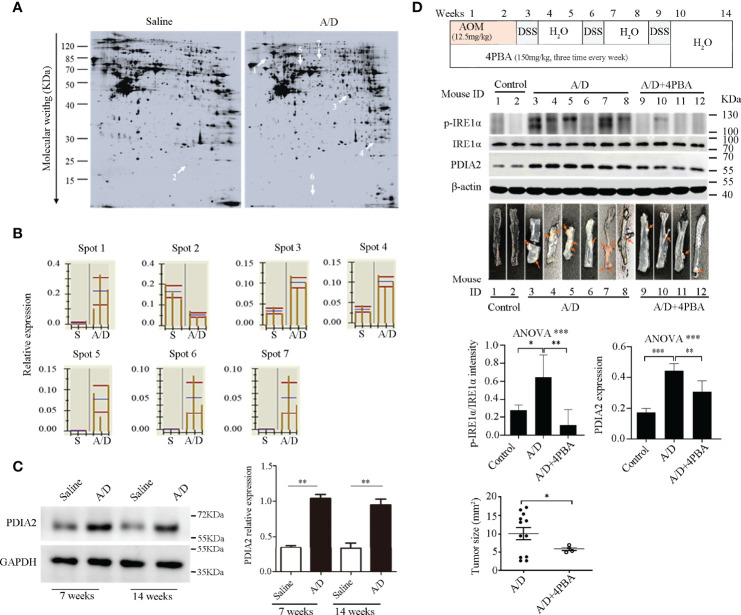
Identification of differentially expressed proteins by proteomics in response to AOM/DSS treatment. **(A)** Display of colon mucosa proteins on two-dimensional electrophoresis gels after saline or AOM/DSS treatment for 7 weeks; the differentially expressed proteins were specified by arrows. **(B)** Quantitative analysis of the 7 differentially expressed proteins by ImageMaster platinum 6.0 software (GE Healthcare, USA). S: saline, A/D: AOM/DSS. Proteins with greater than two-fold difference (*p* < 0.05) were selected; one presentative data of three individual experiments was shown. **(C)** Protein disulfide-isomerase A2 (PDIA2) overexpression in AOM/DSS-treated colon tissues was verified by Western blot. Densitometric analysis of PDIA2 expression was shown in the right panel. **(D)** Flow chart of 4-phenylbutyric acid (4-PBA) treatment was shown in the upper panel. The changes of the IRE1α phosphorylation status (p-IRE1α), PDIA2, and IRE1α by Western blot analysis were shown as the mean ± SD of individual band intensity in each group in the middle panel, and the counts and sizes of tumors after the treatment of AOM/DSS or the combination of AOM/DSS with 4-PBA were shown in the bottom panel. The upregulations of p-IRE1α and PDIA2 were inhibited by 4-PBA (upper-right panel). Tumors inside the images were indicated with orange arrows; data were shown as the mean ± SD of two individual experiments. *, **, and *** represent *p <*0.05, <0.01, and <0.001, respectively.

A colon cancer animal model was established in C57BL/6N mice by the intraperitoneal injection of a colon-specific carcinogen azoxymethane (AOM), and the oral administration of a nongenotoxic sulfated polysaccharide dextran sodium sulfate (DSS) according to a protocol approved by the Ethics Committee of Shanghai Public Health Clinic Center (2014-A032-02). Six to 8-week-old male mice from the Shanghai Public Health Clinical Center were divided into AMO/DSS treatment and control groups (n=10/group). Mice were intraperitoneally injected one dose of AOM (12.5 mg/kg in saline) or saline (0.2 ml) and followed by feeding DSS (W/V = 2.5% in drinking water) or drinking water, respectively, as schemed in **Figure 1A**. In brief, after 1-week free access to water and food, all mice in the AOM/DSS group were given a single dose of AOM (12.5 mg/kg body weight) and were treated in cycles with 2.5% DSS for 7 days and drinking water for 2 weeks till the 6th or 9th week; animals were then free of drugs till 7 or 14 weeks. The control animals were given a single dose of saline injection in correspondence to AOM injection and were followed by drinking water all the time. In specified experiments, animals were treated with AOM/DSS drinking water or the combination of AOM/DSS drinking water and intraperitoneal injection of 4-phenylbutyric acid (4-PBA) (150 mg/kg) three times every week for 9 weeks (on the first day, 4-PBA was given before AOM for 1 h) as schemed in **Figure 2D**. Changes in body weight were recorded every other day, and tumors were counted and colon mucosa were collected for Western blot analysis at 14 weeks. Tumor development was monitored by colon capsule endoscopy (CCE) at the 7th and 14th weeks after the initiation of cancer induction. Animals were killed after each endoscopy, tumor sizes were measured using a vernier caliper, and tumor development was confirmed by pathological examination. Colon was collected for mucosa separation, proteomics, metabolomics, and Western blot analysis.

### Mucosal Separation and Purification

Colorectum was collected at the first day of the 7th and 14th weeks after the initiation of AMO/DSS treatment; colon mucosa was separated according to a protocol described elsewhere (29). Briefly, colorectum was cut open longitudinally and was cleaned with saline for 3 times. Colon mucosa was scraped with a histological glass slide under a dissecting microscope and collected in 15 ml Eppendorf tube. One part of mucosa was immediately fixed in 10% formalin, embedded in paraffin, sectioned, and stained with hematoxylin–eosin staining for histology analysis using an Olympus BX40 light microscope equipped with a logenE PAS9000 (Olympus Corporation, Tokyo, Japan). Another part of the mucosa sample was stored at -80°C for proteomic analysis.

### Two-Dimensional Gel Electrophoresis (2DE)

Colon mucosa tissues were collected at the 7th week after cancer induction and lysed by a RIPA lysis buffer. An equal amount of proteins was separated by 2DE as described elsewhere (30, 31). Briefly, 1.0 mg of proteins was resuspended in a buffer containing 8 M urea, 2 M thiourea, and 4% (w/v) 3-[(3-Cholamidopropyl)dimethylammonio]propanesulfonate (CHAPS), and were loaded in 18-cm, pH 3–10 non-linear immobilized pH gradient (IPG) dry strips [General Electric Company (GE), Chicago, USA). Isoelectric focusing (IEF) was performed in an IPGphor isoelectronic focusing system (GE) up to 52.1 KVh at 20°C. After being equilibrated in 1% (w/v) dithiothreitol (DTT) for 20 min and 2.5% (w/v) iodoacetamide (IAA) for another 20 min, the strips were separated on 12.5% polyacrylamide SDS-PAGE gel at 15°C with a constant current (40 mA) in a Bio-Rad Protein II electrophoresis apparatus (Bio-Rad Company, California, USA). Gels were stained with Coomassie blue. Protein patterns from three replicates were compared with the Imagemaster platinum 6.0 software (GE Healthcare, Chicago, USA). To correct for variability due to staining, and to reflect the quantitative variations of the protein spots, the individual spot volume was normalized by dividing the OD value with the total OD values of all the spots in the gel. For difference analysis, the threshold was defined as the significant change in the spot volume with at least a two-fold difference between control and AOM/DSS groups (*p <*0.05). Furthermore, a manual check followed to make sure that the differential spots were detected at least in two replicates.

### Protein Identification by Mass Spectrometry

Differentially expressed spots in SDS-PAGE gels were excised, digested in 12.5 ng/μl of trypsin (Roche Molecular Biochemicals, Indiana, USA) in 25 mM ammonium bicarbonate (pH 8.5) at 37°C for overnight, and identified by liquid chromatography–mass spectrometry (LC-MS) (30). Ten microliters of the peptide mixture were analyzed by an esquire high-capacity ion trap (HCT) mass spectrometer (Bruker, Karlsruhe, Germany). After desalination by C18 μ-precolumn (300 μm id × 5 mm, 5 μm, PepMap™, LC Packings, Amsterdam, the Netherlands), the peptides were separated by C-18 reversed-phase nanocolumn (75 μm id × 15 cm length, 3 μm, PepMap™, LC Packings, Dionex, Sunnyvale, CA, USA) through a 3%–50% continuous acetonitrile gradient at 300 nl/min. The eluted peptides were on-line injected to a PicoTip emitter nanospray needle (New Objective, Woburn, MA, USA) for HCT mass spectrometer detection.

The MS/MS data were input to the Biotools 3.2 program (Bruker Daltonics, Billerica, MA, USA) to search against International Protein Index (IPI) database identification. Search parameters were set as follows: enzyme, trypsin; allowance for up to one missed cleavage peptide; mass tolerance, 1.2 Da and MS/MS mass tolerance, 0.6 Da; fixed modification parameter, carbamoylmethylation (C); variable modification parameters, oxidation (at Met); auto hits allowed (only significant hits were reported); and the result format as a peptide summary report. Proteins were identified on the basis of peptides whose ion scores exceeded the threshold (*p* < 0.05), which indicates identification at the 95% confidence level for these matched peptides. Proteins identified by more than 4 peptides were accepted without manual checking. For proteins identified with less peptides, manual checking was performed to make sure that at least one peptide has four or more continuing y-or b-series ions (e.g., y4, y5, y6, and y7).

### Metabolite Quantification by Mass Spectrometry

Colon tissues (approximately 10 mg) were homogenized in normal saline (100 µl) with 60 Hertz for 60 s and cooled on ice for 60 s. After 3 homogenization–cooling cycles, the homogenates were precipitated with 30% (w/v) trichloroacetic acid containing the internal standard U-13C6 glucose (10 µg/ml, 300 µl), and were followed by incubation at 4°C for 30 min and centrifugation with 10,000 g for 10 min at 4°C. The supernatant (5 µl) was loaded onto an ultra-high-performance liquid chromatography/tandem mass spectrometry (UHPLC-MS) (Waters Acquity UPLC system (Waters Corporation, Milford, CT, USA) and an AB Sciex Triple Quad 5500 mass spectrometer (Applied Biosystems/AB SCIEX, Boston, MA, USA) for metabolite quantification. The analysts were separated by an Acquity UHPLC HSS T3 1.8-µm column (2.1 × 100 mm, Waters) with the mobile phase A of 10 mmol/L ammonium format and the mobile phase B of acetonitrile (ACN).

Mass spectrometry detection was performed using multiple reaction monitoring (MRM) in a negative-ion mode. The optimal MS-operating conditions were as follows: spray voltage, −4,500 V; temperature, 500°C; collision gas (CAD), medium; curtain gas pressure (CUR), 40 psi; ion source gas 1 and ion source gas 2, 60 psi; entrance potential, 10 eV; and collision cell exit potential, 10 eV. The analytes were quantified by the MRM mode using suitable MS conditions as shown in **Supplement Table S2**.

### Western Blotting Analysis and Coimmunoprecipitation

Fifty micrograms of protein extracts from human and mouse colon tissues or cells were separated by electrophoresis in SDS-10% polyacrylamide gel and transferred to a polyvinylidene fluoride (PVDF) membrane (Millipore Company, MA, USA) as described previously (30, 31). After blocking in 10% defatted milk for 30 min, blots were incubated overnight at 4°C with antibodies against PDIA2 (1:1,000), IRF1α (1:500), p-IRF1α (1:500), β-actin (1:1,000), or GAPDH (1:1,000) or total OXPHOS (1:250) primary antibodies. After three washes with TBS-Tween-20, blots were incubated for 1 h at 20°C with an HRP-conjugated secondary antibody (dilution at 1: 5,000). After further washes, the immune complexes were shown by enhanced chemiluminescence and detected by the chemiluminescence imaging system of ChemiScope 5300 X-rays (Clinx Science Instruments, Shanghai, China). Each experiment was repeated three times.

For the coimmunoprecipitation of PDIA2 with mitochondrial complex components, the total cell lysates of HT-29 colon cancer cells (containing 600 µg total proteins) were precleaned with protein A/G PLUS-agarose (Santacruz) and were followed by incubation with 4 µg of either a rabbit polyclonal antibody against human PDIA2 or rabbit IgG at 4°C overnight in a rotation platform. Protein A/G PLUS-agarose was added into precipitations and rotated at 4°C for 1 h. After 3 washes with a lysis buffer, the immune complexes were boiled to release them from agarose beads and were used for proteomic profiling or immune blotted with the total OXPHOS rodent WB antibody cocktail.

### Tissue Array and Immunohistochemistry

A chip for a tissue array was made by Shanghai Outdo Biotech CO., LTD (Shanghai, China) using the above 90 pairs of colon cancer and adjacent tissues. Immunohistochemistry (IHC) analysis was performed using a commercial IHC kit (Shanghai Outdo Biotech CO., LTD, Shanghai, China) with the PDIA2 antibody at a dilution of 1:300 according to the manufacturer’s directions and developed with a liquid 3, 3’-diaminobenzidine (DAB; DAKO Company, Shanghai, China). Sections were counterstained with hematoxylin-exon, dehydrated, mounted in Permount (Thermo Fisher Scientific, Waltham, MA, USA), and scanned with Scanscope XT (Aperio Technologies, Inc, CA, USA). The antigen density was analyzed by Aperio ImageScope software (Aperio Technologies). Samples without visible colon mucous membranes were excluded from the analysis.

### Real-Time PCR Analysis

The mRNA expression of PDI isoforms (PDIA1, 2, 3, 4, 5, and 6) from surgical colon cancer and their adjacent tissues (n = 6 pairs) were detected by real-time PCR. Total human colon tissue mRNA was extracted using a TRIzol reagent (Life Technologies, USA), and was subjected to reverse transcription using a Moloney murine leukemia virus reverse transcriptase (Promega). Real-time RT-PCR was carried out using a TransGen qPCR kit. The specific primers were shown in **Supplement Table S3**.

### Protein Disulfide Isomerase–Associated 2 RNA Interference

The knockdown expression of the PDIA2 gene was conducted using shRNA interference strategy. Briefly, three shRNA oligonucleotides specific for the PDIA2 gene (named C1, C2, and C3) were designed based on the gene sequence from the Genebank accession number (Genebank: NM_006849) and chemically synthesized by Shanghai Genechem Co., Ltd. (Shanghai, China). The shRNA sequences and their targeted sequences were shown in **Supplement Table S4**. Infectious lentiviral particles were constructed by introducing gene- specific shRNAs into plasmid GV248 containing EGFP Flag and Amp^r^ selection genes and were used for HT29 cell infection. GV248 plasmid–transfected cells (named C0) served as control. Stable transfection of lentivirus was established in a selection medium containing 100 μg/ml ampicillin. The cell viability was detected by a cell counting Kit-8 (CCK8) according to manufacturer’s instruction. Cell apoptosis was analyzed by flow cytometry using a flow cytometer (Beckman, Florida, USA).

### Immunofluorescence Analysis

To detect the subcellular location of PDIA2, HT29 colon cancer cells and CCD-18Co normal human colon cells were seeded into Lab-Tek cell culture wells (155411, Thermo Scientific) for 12 h; the cell culture medium was removed, and prewarmed (37°C) staining solution containing the Mito-Tracker probe was added into wells. After 30-min incubation at 37°C, cells were washed three times with PBS, fixed with 4% paraformaldehyde, and incubated with a PDIA2 antibody overnight at 4°C. After 3 washes with PBS, cells were incubated with Alexa Fluor488 goat-anti-rabbit IgG (H+L) for 1 h and followed by nuclear counterstaining for 5 min with DAPI (5 μg/ml, Sigma). Slides were then mounted with glycerol and analyzed using a Leica TCS SP5 microscope (Leica) with LAS AF Lite 4.0 image browser software.

### Subcutaneous Tumor Xenograft Animal Model

Four-to-five-week-old male nude mice were purchased from the Shanghai Public Health Clinical Center and maintained at a specific pathogen-free (SPF) room according to a protocol approved by the Ethics Committee of Shanghai Public Health Clinical Center (No. 2018-A025-01). Mice were grouped into HT29 [wild-type (Wt), n=6], C0 (scramble, n=6), and C3 (PDAI2 shRNA, n=7). Each mouse was subcutaneously injected in the flank with 10^6^ cells in 100 μl of saline (32). Xenograft tumors in all groups became visible within 2 weeks after injection. Since then, the tumor size was measured once every 3 days till 28 days after subcutaneous cell injection. Mice were then anesthetized, weighted, and euthanized; tumors were scraped and weighed.

### Energetic Assay

Mitochondrial respiration was assessed using a Mito stress kit. In brief, Wt or PDIA2-knockdown HT29 cells (2 × 10^5^/well) were set for overnight in a 24-well microplate (Agilent Technologies, CA, USA) in a modified Mccoy’s 5A medium supplemented with 10% fetal bovine serum (FBS). Cells were washed with a basal assay medium (pH 7.4, Invitrogen, CA, USA) and preincubated in a basal assay medium for 1 h at 37°C in a CO_2_-free incubator. Then 10-fold concentrated compounds (Agilent Technologies) of oligomycin, carbonyl cyanide-p-trifluoromethoxyphenylhydrazone (FCCP), or a mixture of rotenone and antimycin A were loaded into a sensor cartridge to produce the final concentrations of 1 μM, 1.5 μM, 100 nM, and 1 μM, respectively. After a 30-min calibration of the XFe sensor with the pre-incubated sensor cartridge, the cell plate was loaded into the XFe-24 Extracellular Flux Analyzer (Agilent Technologies); the mitochondrial oxygen consumption rate (OCR) was analyzed under basal conditions and followed by a sequential injection of the complex inhibitors oligomycin, FCCP, and the mixture of rotenone and antimycin A. Data were analyzed using XFe software (Agilent Technologies) and normalized with protein loaded in each well. Four replicates of each sample were analyzed.

The cellular glycolytic capacity was evaluated by the extracellular acidification rate (ECAR) with the glycolysis stress kit according to the manufacture's protocol. The background glycolysis was determined by incubating cells in an assay medium in the absence of glucose or pyruvate; the basal ECAR was measured after adding glucose (10 mM in final). the difference between the basal ECAR and the ECAR after the addition of oligomycin (1 µM in final) was determined as the glycolytic capacity, and the difference between oligomycin-induced ECAR and the hexokinase inhibitor 2-deoxy-D-glucose (2-DG, 50 mM in final)–conducted ECAR was defined as a glycolytic reserve.

For a metabolic assay on subcutaneous tumor cells, the subcutaneous tumor tissues from nude mice were cut into 1–3 mm^3^ pieces using a sterile scalpel scissor and were followed by enzymatic digestion for 5 min at room temperature in Eagle’s minimum essential medium containing collagenase type I (200 units/ml) and DNase (270 units/ml) (Sigma, St. Louis, MO, USA). Single-cell suspensions were prepared by passing the digestion mixture through a 4-layer sterile gauze. Cells were washed 3 times in a serum-free medium and resuspended in 24-well Seahorse XF cell culture plates for overnight in Eagle’s minimum essential medium with 10% fetal bovine serum and were subject to glycolysis stress and mitochondrial stress assays.

### Statistical Analysis

A two-group *t* Test software packed in Imagemaster software was used for the analysis of protein spot density in 2DE. GraphPad Prism software (vision 5) was used for the analysis of mouse body weight, tumor size, WB, real-time PCR, metabolite quantification, and immunohistochemistry data. The difference between the two groups were analyzed by a two-tailed unpaired *t-* test except the comparisons of WB and real-time PCR from 6 pairs of clinical colon tissues by the two-tailed paired t-test. Significant differences among multiple groups were determined by one-way ANOVA analysis. *P*-values less than 0.05 were considered as statistically significant.

## Results

### Animal Model of Malignant Transformation of Chronic Bowel Inflammation

To explore the molecular mechanisms of inflammation to cancerous transformation, an animal model for chronic bowel inflammation to colon cancer transformation has been generated and cancer development has been verified as schemed in **Figures 1A, B**. Under endoscopy, in contrast to the pink-look and smooth colon mucosa at the 7th and 14th weeks in saline control mice, spotting or seriously spread bleeding, edema with mild erosion, and the protuberances of colon mucosa were observed in the descending colon segment in AOM/DSS-treated mice. Of them, 5 of 10 mice have developed a total of 5 tumors with the mean tumor size of 6.99 ± 1.62 mm^2^ at the 7th week, and all 10 mice have developed a total of 38 tumors with the mean tumor size of 9.35 ± 3.87 mm^2^ that were large enough to occupy more than half colon cavity at the 14th week (**Figure 1C**; **Supplement Table S5**). The center area of protuberance appeared pale due to anemia but red in the peripheral area due to hyperemia (**Figure 1D**). An anatomical exam found protuberances not only in descending but also in transverse and ascending colon segments in all AOM/DSS-treated animals (**Figure 1E**). Cancer development was confirmed by histological changes typical to colon cancer characterized by irregular submucosa arrangement, mucosa epithelial dysplasia, immune cell infiltration, lymphatic follicle formation, and cells with a deep-stained large nucleus (**Figure 1F**).

AOM/DSS treatment caused hyporexia, leading to the body weight loss of animals approximately 4% at the 2nd week, 11% at the 7th week, and 20% at the 14th week in comparison with that of the saline control group (**Supplementary Figure S1**).

### Identification of Differentially Expressed Proteins in Response to AOM/DSS Treatment

To explore the key molecules in promoting chronic colitis to cancerous transformation, we explored the differential protein expression of colon epithelia between saline and AOM/DSS-treated animals using a two-dimensional polyacrylamide gel electrophoresis (2D-PAGE) assay. Colon mucosa were collected at the 7th week after treatment and were confirmed to exclude any muscle and film layer by a histology exam (**Supplementary Figure S2**). The lysates of mucosa tissues were loaded and electrophoresed on the 2D-PAGE gel, and the protein display was analyzed using the Imagemaster software (**Figure 2A**). In total, 1,067 ± 48 and 1,077 ± 59 protein spots were displayed in gels from saline and AOM/DSS-treated animals, respectively, and six spots were upregulated and one spot was downregulated for more than twofold in the AOM/DSS group in comparison with those in the saline-treated group (**Figure 2B**). Their corresponding proteins were identified based on the protein database of the MS/MS spectrum and were shown in **Table 1** and **Supplementary Table S6** (confidence ≥95%). The increased proteins were the PDI-associated 2 (PDIA2, an ER chaperone protein family member), heterogeneous nuclear ribonucleoprotein A/B (HNRNPAB, ssRNA-binding protein), beta-globin (a subunit of hemoglobin), keratin 8 (KRT8), pyrroline-5-carboxylate reductase 2 (PYCR2, proline synthase), and Vault structure regulator (Mvp). Among them, PDIA2 stood out of others and expressed approximately 7-fold more in AOM/DSS-treated than that in saline-treated colon mucosa (**Figure 2B**). The increased PDIA2 protein was further confirmed by Western blot analysis (**Figure 2C**).

**Table 1:** The differentially expressed proteins between saline and azoxymethane/dextran sodium sulfate–treated colon mucosa.

Spot	Accession number	Protein name	Score	PI	Matched peptides	MW	Fold change*	Molecular function	Biological process
1	IPI00347894	Protein disulfide-isomerase A2	109	4.7	6	58565	↑7.37	Enzyme; binding	Cell redox homeostasis; apoptotic signaling pathway
2	IPI00762198	Beta-globin	47	9.1	3	16280	↓2.05	Binding; enzyme	Oxygen transport
3	IPI00117288	Heterogeneous nuclear ribonucleoprotein A/B	115	8.7	6	30926	↑2.19	Binding	Epithelial-to- mesenchymal transition
4	IPI00762198	Beta-globin	55	9.1	3	16280	↑2.22		
5	IPI00322209	Keratin, type II cytoskeletal 8	203	5.6	12	54531	+∞	Binding; structural molecular activity	Cell differentiation
6	IPI00123278	Pyrroline-5-carboxylate reductase 2	50	7.7	1	33980	+∞	Binding; transporter	Oxygen transport
7	IPI00111258	Mvp major vault protein	49	5.4	3	97083	↑2.07	Binding	Erythroblastic oncogene B (ERBB) signaling pathway

PI, propidium iodide. ↑, up-regulated in AOM/DSS treated group compared with the control; ↓, down-regulated in AOM/DSS treated group compared with the control; +∞, only detected in AOM/DSS treated group.

As PDIA2 is one of the ER chaperone protein family members that are usually upregulated in response to ER stress, we further confirmed if AOM/DSS treatment induces ER stress by analyzing the phosphorylation status of inositol-requiring transmembrane kinase/endoribonuclease 1α (IRE1α) (another biomarker of ER stress) in colon mucosa. AOM/DSS treatment significantly increased the level of IRE1α phosphorylation, which was significantly attenuated by the administration of the ER stress inhibitor 4-phenylbutyric acid (4-PBA). Consistent with the change of IRE1α phosphorylation, AOM/DSS administration also increased the PDIA2 protein level that was restored to the basal level by 4-PBA treatment (**Figure 2D**). To determine if the inhibition of ER stress can impede the malignant transformation of chronic colitis, we found that animals treated with the combination of AOM/DSS plus 4-PBA developed a total of 4 tumors in 4 animals with the tumor size 5.85 ± 0.95 mm^2^, in contrast to the total of 13 tumors in 6 animals with the tumor size 9.66 ± 5.65 mm^2^ (*p* = 0.0367, **Figure 2D**). These data collectively suggested a role of ER stress and its associated PDIA2 overexpression in inflammation to cancerous transformation.

### Communications of Overexpressed Protein Disulfide Isomerase–Associated 2 With Metabolic Pathways in Response to AOM/DSS Treatment

**Figure 3:**
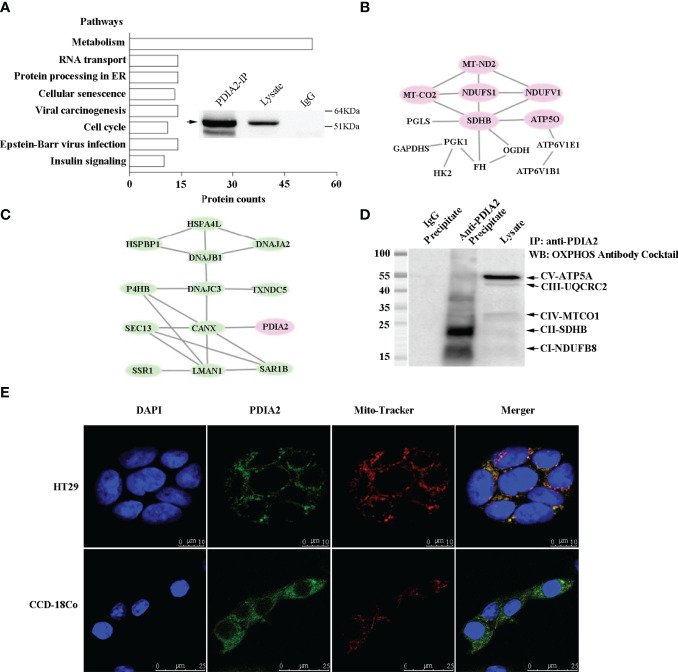
Coimmunoprecipitation–mass spectrum (Co-IP/MS) analysis of PDIA2-interacting proteins from the HT-29 cell lysate. PDIA2 coimmunoprecipitated with 420 proteins, and the KEGG enrichment was analyzed by STRING software (version 11.0). **(A)** The overall pathways that were networked by PDIA2. The metabolic pathways were mostly enriched, in which 53 proteins interact with PDIA2. The inset showed the efficient immunoprecipitation of PDIA2 by a gene-specific antibody. IP, immunoprecipitation. **(B)** Part of protein–interaction network in metabolic pathways; proteins involved in mitochondrial functions were specified in red. **(C)** The protein–interaction network in endoplasmic reticulum. **(D)** Western blot analysis of the interaction between PDIA2 and electron transport complexes; labels on the right indicate the subunit of each complex. **(E)** Confocal microscopy analysis of mitochondrial translocation of PDIA2 in HT-29 colon cancer cells and CCD-18Co human normal colon cells.

ER stress broadly communicates with multiple cellular activities. To explore the association of PDIA2 overexpression with the changes of cellular signaling pathways in response to AOM/DSS treatment, PDIA2-interacting proteins in HT29 colon cancer cells were detected by PDIA2 coimmunoprecipitation (CO-IP)–mass spectrum analysis. PDIA2 was efficiently immunoprecipitated by the anti-PDIA2 antibody (**Figure 3A** insert) and a total of 420 proteins were coimmunoprecipitated with PDIA2 (**Supplement Table S7**). KEGG pathway enrichment analysis showed the interactions of PDIA2 with proteins in 8 signaling pathways (*p* < 0.05), including insulin signaling, Epstein–Barr virus infection, cell cycle, viral carcinogenesis, cellular senescence, protein processing in ER, RNA transport, and metabolism. In particular, PDIA2 coimmunoprecipitated with 53 proteins in metabolic pathways in contrast with approximately 10 proteins in the other 7 pathways (**Figure 3A**; **Supplement Table S8**), emphasizing the role of PDIA2 in regulating metabolic modifications during inflammation to cancerous transformation.

To support the role of PDIA2 in metabolic pathways, further analysis on the protein–protein network revealed that PDIA2 coprecipitated with metabolic proteins key for the glycolytic pathway (HK2, PGK1, and GAPDHS), pentose phosphate pathway (PGLS), Krebs cycle (SDHB, OGDH, FH), and electron transport chain (NDUFS1, NDUFV1, MT-ND2, SDHB, MT-CO2, and ATP5O) (**Figure 3B**; **Supplement Table S8**). Remarkably, the AOM/DSS-induced ER stress appeared to predominantly affect mitochondrial respiration. Six key proteins from complex I to complex V across electron transport chain were networked together; three of them (NDUFS1, NDUFV1, and MT-ND2) are the subunits of complex I. In addition, probably as a consequence of metabolism remodeling, key proteins for cellular acidification (ATP6V1B1 and ATP6V1E1) were also networked together with metabolic proteins, indicating an increase in glycolysis. Other PDIA2-interacting proteins included 14 ER stress–associated proteins functioning in protein folding [P4HB, DNAJC3, HSPBP1, DNAJB1, DNAJA2, and HSPA4L), protein secretion processing (LMAN1, SAR1B), misfolding protein degradation (DERL1), antioxidant (TXNDC5), and calcium homeostasis (CANX) (**Figure 3C**; **Supplement Table S8**)], demonstrating a typical unfolded protein response (UPR) in response to AOM/DSS stimulation.

To confirm the interaction of PDIA2 with any component of the mitochondrial oxidative phosphorylation complexes in colon cancer cells, HT-29 cell lysates were immunoprecipitated with an anti-PDIA2 antibody or control IgG. The immunoprecipitates were probed with an antibody cocktail against proteins representing the five mitochondrial respiratory complexes, i.e., CI-20kDa subunit NDUFB8, CII-30kDa SDHB, CIII-48kDa Core protein 2 (UQCRC2), CIV-40kDa subunit I (MTCO1), and CV-55kDa alpha subunit ATP5A. The coimmunoprecipitation assay demonstrated that PDIA2 interacts with the component of complexes I and II, respectively (**Figure 3D**). Furthermore, immunofluorescence staining in colon cancer cells indicated PDIA2 translocation into perinuclear mitochondria by showing the merging of its staining fluorescence with that of mitochondrial probe Mito-Tracker Deep Red FM. However, the colocalization of PDIA2 with Mito-Tracker was not observed in CCD-18Co human fibroblast cells isolated from normal colon tissue (**Figure 3E**). These data collectively suggested a role of PDIA2-associated metabolic reprogramming by modifying the functions of the electron transport chain during inflammation to cancerous transformation.

### Downexpression of Protein Disulfide Isomerase–Associated 2 Restores Metabolic Homeostasis in Colon Cancer Cells

**Figure 4:**
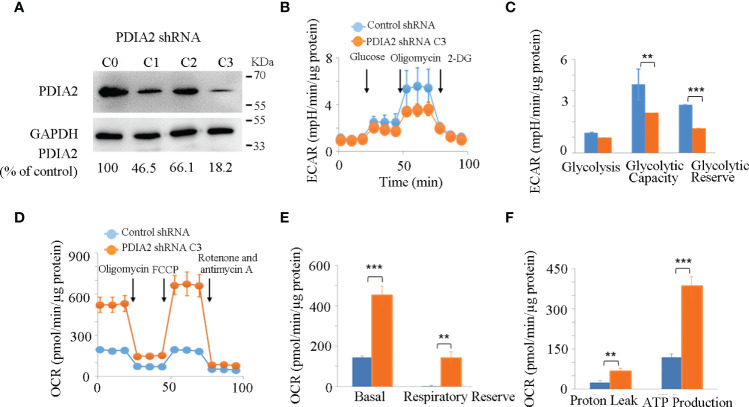
Knockdown expression of PDIA2 restored metabolic homeostasis in colon cancer cells. **(A)** The efficiency of PDIA2 knockdown by shRNA strategy. C0 represents control knockdown cells; C1, C2, and C3 indicate the subclone cells with PDIA2 knockdown. C3 was selected for subsequent studies. **(B)** Glycolysis stress assay of control and PDIA2-knockdown cells using Seahorse extracellular flux (XFe-24) analyzer. 2-DG: 2-Deoxy-D-glucose. **(C)** Comparison of basal glycolysis (extracellular acidification rate, ECAR), glycolytic capacity, and reserve between control and PDIA2-knockdown cells. **(D)** Mitochondrial stress assay of control and PDIA2-knockdown cells using a Seahorse extracellular flux (XFe-24) analyzer. FCCP: carbonyl cyanide-4 (trifluoromethoxy) phenylhydrazone. **(E)** Comparison of basal mitochondrial respiration (oxygen consumption rate, OCR) and respiratory reserve between control and PDIA2-knockdown cells. **(F)** Comparison of proton leak (oxophosphorylation uncoupling) and ATP production between control and PDIA2-knockdown cells. ** and *** represent *P* < 0.01 and 0.001, respectively.

To confirm the results of KEGG analysis that highly indicated the association of AOM/DSS-induced PDIA2 overexpression with metabolism modifications, particularly the modification of mitochondrial respiration, PDIA2 expression was partially inhibited in HT-29 colon cancer cells by gene-specific shRNA nucleotide sequences. Three subclones (C1, C2, and C3) were established and respectively expressed over 50%, 30%, and 80% less of PDIA2 protein in comparison with that in control shRNA subclone (C0) cells (**Figure 4A**); thus, subclone C3 cells were selected for subsequent studies. To determine if PDIA2 knockdown affects the levels of ER stress and electron transport complex expression, the protein levels of IRE1α and p-IRE1α were detected by Western blot and found that they were reduced, whereas the level of complex II was increased in C3 cells in comparison with that in Wt HT29 cells (**Supplementary Figure S3**).

Hyperglycolysis is a typical signature of both inflammatory and cancer cells; we then firstly compared the real-time kinetics of glycolysis by measuring the extracellular acidification rate (ECAR) in control and PDIA2-knockdown cells using the glycolytic stress assay. Although both control and PDIA2- knockdown cells showed a comparable basal glycolysis, PDIA2 knockdown significantly repressed the glycolytic stress response by reducing the glycolytic capacity (the maximum rate of conversion of glucose to pyruvate or lactate) and glycolytic reserve (the difference between the glycolytic capacity and basal glycolysis in the presence of glucose) in comparison with those in control cells (**Figures 4B, C** **)**.

The hyperglycolytic activity in cancer cells usually results from the dysfunction of mitochondrial respiration. We thus tested the effect of PDIA2-knockdown expression on mitochondrial respiration by measuring the mitochondrial oxygen consumption rate (OCR) in control and PDIA2-knockdown cells using the mitochondrial stress assay. In contrast to the repression of glycolysis, PDIA2 knockdown surprisingly doubled the basal OCR of the control cells and significantly increased the respiratory reserve (the difference between the maximum OCR and basal OCR) (**Figures 4D, E** **)**. As a consequence of the increase in mitochondrial OCR, the coupling of oxidative phosphorylation (proton leak) and ATP production were also parallelly enhanced in PDIA2-knockdown cells (**Figure 4F**). These observations collectively indicated that the overexpression of PDIA2, a typical indicator of ER stress, plays a role in modifying mitochondrial respiration, leading to an energetic switch from mitochondrial respiration to cytosolic glycolysis that favors cancer development.

### Repression of Endoplasmic Reticulum Stress Restores Metabolic Homeostasis in AOM/DSS-Induced Colon Cancer Cells

**Figure 5:**
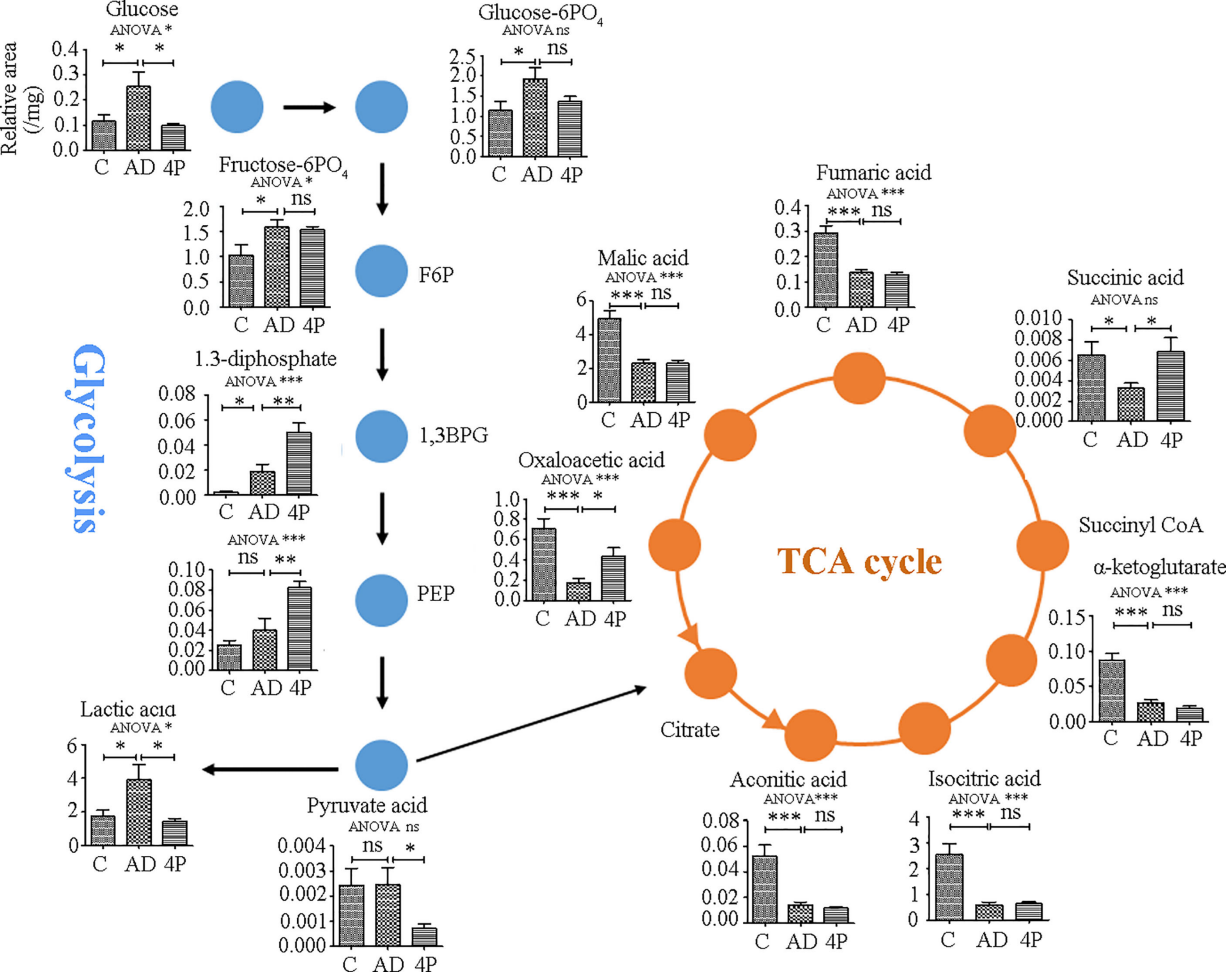
Metabolite changes induced by AOM/DSS and 4PBA treatments in a mice model. Mice were treated with saline, AOM/DSS, or AOM/DSS plus 4PBA for 7 weeks. Colon tissues were collected, and the metabolites involved in glycolysis and the TCA cycle were quantified by LC-MS/MS. The levels of metabolites were expressed as relative signal intensity (analyst to internal standard)/mg tissue. C: saline treatment; AD: AOM/DSS; 4P: AOM/DSS and 4PBA treatment. Data were shown as mean ± SD, n=8 in each group. * *p* < 0.05, ** *p* < 0.01, *** *p* < 0.001. ns, not significant.

To translate the cell model data into *in vivo* animal models, we compared the changes of the metabolites of energy-producing pathways (glycolysis and the citric acid cycle) in response to AOM/DSS and the combination of AOM/DSS and 4PBA by UHPLC-MS/MS. As shown in **Figure 5** and **Supplement Table S9**, AOM/DSS treatment significantly increased the levels of the compounds of the glycolysis pathway, including glucose, glucose-6-phosphorate, fructose-6-phosphotate, 1,3-bisphosphoglycerate, and lactate, but did not change the levels of phosphoenolpyruvate and pyruvate. 4PBA therapy restored the AOM/DSS-mediated increases of glucose and lactate and furthermore increased the levels of 1,3-bisphosphoglycerate and phosphoenolpyruvate but significantly reduced the level of pyruvate. These changes of glycolytic compounds suggested that AOM/DSS activates the glycolytic activity, which is potentially restored by 4PBA therapy. Remarkably, the increases in 1,3-bisphosphoglycerate and phosphoenolpyruvate by 4PBA implicate the activation of gluconeogenesis, and the reduction of pyruvate by 4PBA suggests the homeostasis restoration of glycolysis and oxidative response.

To support the switch of oxidative metabolism to glycolysis by AOM/DSS treatment, all the detected metabolites of the tricarboxylic acid cycle (TCA) were significantly reduced, including aconitic acid, isocitric acid, α-ketoglutarate, succinic acid, fumaric acid, malic acid, and oxaloacetic acid. 4PBA therapy restored the levels of succinic acid and oxaloacetic acid; however, it did not change the levels of others (**Figure 5**), highlighting the complexity of the TCA cycle as an exchange hub of metabolites from different metabolic pathways.

### Downexpression of Protein Disulfide Isomerase–Associated 2 Inhibits Subcutaneous Tumor Growth

**Figure 6:**
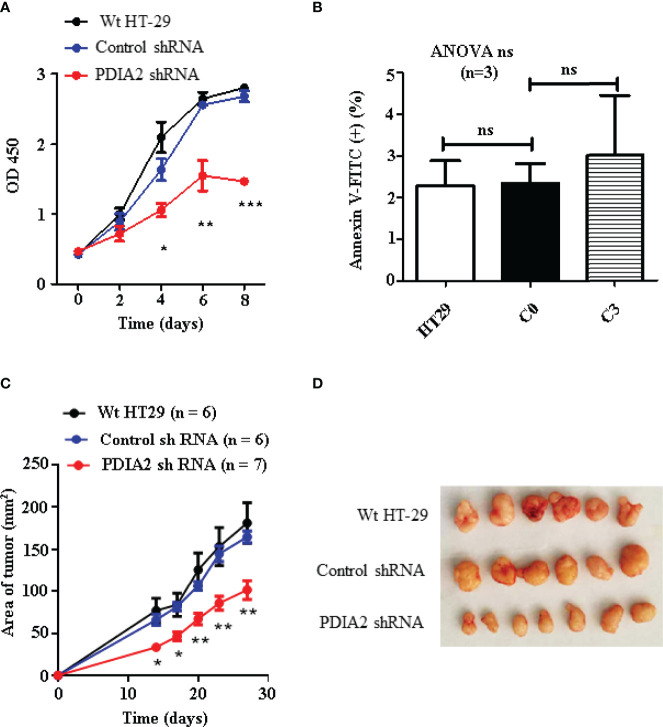
Knockdown expression of PDIA2 inhibited the growth of the subcutaneous xenograft colon cancer. **(A)** Cell growth analysis of wild-type (Wt), control knockdown and PDIA2-knockdown HT-29 colon cancer cells by Cell Counting Kit-8 assay. **(B)** Apoptotic cells were costained with annexin-FITC and propidium iodide, and were analyzed by flow cytometry. **(C)** The follow-up of the xenograft tumor size by a vernier caliper; data were presented as mean ± SD. **(D)** The xenograft tumors at 27 days after cell transplantation. *, **, and *** represent *p* < 0.05, 0.01, and 0.001, respectively. ns, not significant.

To determine if PDIA2 overexpression affects the proliferation of cancer cells, we compared the cell growth rate of Wt, control shRNA (C0)- and PDIA2 shRNA (C3)-transfected HT-29 cells by the CCK8 assay. Both Wt and control shRNA cells quickly entered the logarithmic growth phase at day 2 and turned into the plateau growth phase at day 6 after the initiation of the cell culture. In contrast, PDIA2-knockdown cells displayed a significantly sluggish growth rate since day 2, peaked at day 6, and declined at day 8. the peak growth rate of PDIA2-knockdown cells was over 40% lower than that in Wt and control shRNA-transfected cells (**Figure 6A**). The reduction of the growth rate was not resulted from apoptosis by PDIA2 gene-specific shRNA (**Figure 6B** and **Supplementary Figure S4**).

To confirm the role of PDIA2 overexpression in cancer development, we next generated a subcutaneous cancer model in immunocompromised nude mice by transplanting Wt, control shRNA (C0)- and PDIA2 shRNA-transfected (C3) HT-29 colon cancer cells. Subcutaneous cancers became visible at 2 weeks after the transplantation of all types of cells; the tumor sizes developed from Wt (77.0 ± 14.7 mm^2^) and control RNAi cells (65.9 ± 6.7 mm^2^) were comparable but were over 2-fold larger than those developed from PDIA2-knockdown cells (33.3 ± 2.9 mm^2^). Since then, tumors from both Wt and control RNAi cells continued to grow fast in parallel, the tumor size reached 181.4 ± 23.9 mm^2^ and 164.8 ± 6.9 mm^2^, respectively, at day 27 after cell transplantation, in contrast to the tumor size 101.6 ± 11.5 mm^2^ from the PDIA2-knockdown cells (**Figures 6C, D** **)**.

As the cancer growth rate largely depends on glycolysis, we reasoned that the metabolism reprogramming during the continuous cell replications is responsible for the change of PDIA2-knockdown cells from sluggish growth at the early stage to logarithmic growth at the late stage in both the *in vitro* and *in vivo* models due to the incomplete knockdown expression of PDIA2. To confirm this possibility, we compared the differences in mitochondrial respiration and glycolysis between Wt and PDIA2-knockdown tumor cells using Seahorse analysis. Although the maximal OCR of PDIA2- knockdown tumor cells was still higher than that of Wt tumor cells, their ECAR became comparable (**Supplementary Figure S5**). Thus, the knockdown expression of PDIA2 and its associated metabolic reprogramming inhibit cancer cell growth and defer fast cancer development, particularly in the early stage, highlighting a prompting role of PDIA2 in inflammation to cancerous transformation.

### Protein Disulfide Isomerase–Associated 2 Is Overexpressed in Colon Cancer Tissues Rather Than Cancer-Adjacent Tissues

**Figure 7:**
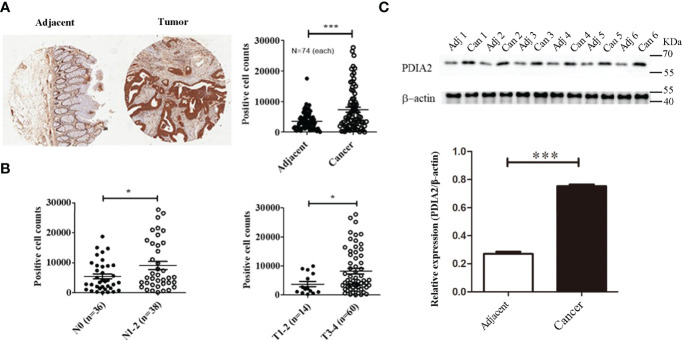
PDIA 2 expression in human colon cancer and cancer-adjacent tissues. **(A)** A representative microarray data of 74 pairs of colon cancer and adjacent tissues (left panel) and PDIA2-positive cell counts by Scanscope XT scanning (right panel). **(B)** The association of PDIA2 overexpression with colon cancer staging and metastasis by a stratified analysis of the microarray results. **(C)** Western blot analysis of PDIA2 expression in human colon cancer and adjacent colon tissues. T: human colon cancer, A: adjacent tissues. * *p* < 0.05, ** *p* < 0.01, *** *p* < 0.001.

To translate the findings of animal and cell model studies to clinic, PDIA2 expression was compared between 74 pairs of qualified tissues from 90 pairs of human colon cancer and cancer-adjacent tissues by gene array analysis (**Supplementary Table S1**). Cancer tissues expressed approximately 2.8-fold more PDIA2 protein than adjacent colon tissues in 61 of 74 pairs of tissues (**Figure 7A**; **Supplementary Figure S6**).

To further reveal the association of PDIA2 over-expression with cancer development stages, cancer tissues were grouped as early stage (T1, T2, and N0) and late stage (T3 and T4, N1 and N2) based on tumor TNM (T: primary tumor; N: regional lymph nodes; M: distant metastasis) classification standard (28). Over-expression of PDIA2 was highly related with late stages (T3 and T4) and lymph node metastasis (N1 and N2) (**Figure 7B**). To confirm the tissue array data, we randomly collected another 6 pairs of colon cancer and their adjacent tissues for Western blot analysis. Both cancer and adjacent tissues expressed PDIA2 protein, and cancer tissues expressed approximately 2.8-fold more PDIA2 protein than their adjacent tissues (**Figure 7C**).

To determine if other isoforms of PDI were overexpressed in colon cancer tissues, the mRNA levels of PDIA1, PDIA2, PDIA3, PDIA4, PDIA5, and PDIA6 in human colon cancer and their adjacent tissues were compared by real-time PCR. Consistent with previous reports (17, 18), PDIA1 and PDIA3 were overexpressed in colon cancer tissues in comparison with that in their adjacent tissues, in addition to the overexpressed PDIA2; however, no statistic changes were detected for PDIA4, PDIA5, and PDIA6 expression (**Supplementary Figure S7** **).**

## Discussion

The current study revealed that the the ER stress–associated overexpression of PDIA2 interacts with the components of electron transport complexes during the inflammatory process of chronic colitis and causes energetics modifications, leading to the switch of mitochondrial respiration to cytosolic glycolysis that favors colon cancer development. These findings collectively highlighted the roles of the ER–mitochondria axis in promoting the malignant transformation of chronic inflammation.

The ER not only functions in proteostasis, lipid biogenesis, calcium, and redox homeostasis but also coordinates energetic fluctuations with metabolic reprogramming processes. Conditions such as an acute proinflammatory cytokine storm produces ER stress that activates an adaptive mechanism of UPR including an increase in the expression of PDIs to cope with the stress by repressing protein translation, degrading unfolded/misfolded proteins, and increasing the capacity of ER to fold proteins; homeostasis is restored upon stress resolution (33). However, chronic inflammation sustains the overexpression of PDIs and accumulates ER stress that fundamentally modifies cellular activities through the physical contacts of ER with mitochondria, plasma membrane, endosomes, and lysosomes and even promotes cancer transformation (34, 35). Our study confirmed the role of ER stress in the malignant transformation of chronic inflammation by showing that the inhibition of ER stress significantly impeded colon cancer development.

PDIs are a group of thiol oxidoreductase chaperones from thioredoxin superfamily and consist of 21 family members with similarities in the amino acid sequence and domain organization but different sets of substrates (14). For example, PDIA1, usually referred to as PDI, is the prototype of this family and functions as a central chaperone for protein folding and a redox regulator *via* isomerization reactions (36); much less is known about the functions of other PDI members. The persistent overexpression of PDIs has been associated with multiple type of cancers and has also been correlated with cancer metastasis, invasion, chemoresistance, and survival rate (17, 36); however, the underlying mechanisms have not been clarified yet. Remarkably, in addition to the ER residents, substantial evidence have indicated that PDIs translocate to extra ER locations such as the cell surface and possibly cytosol and participate in cellular activities such as agonist-triggered NADPH oxidase (Nox) activation, cell migration in vascular cells and macrophages, expansive caliber remodeling during injury repair, and the induction of HIV infection (37); however, how the subcellular PDIs translocation occurs and if these external ER PDIs play roles in cancer development remain unclear.

PDIA2 was originally isolated and characterized as pancreas-specific PDI (PDIp) with amino acid sequence 46% identity and 66% similarity to that of human PDIA1 (38), It plays a similar chaperone role as PDIA1 but has different substrate specificity and is less effective in oxidation/reduction regulation (39, 40), In addition, PDIA2 regulates tissue factor (TF) [a cellular receptor for plasma protease factor VIIa (FVIIa)] polarization in human vascular smooth muscle cells during migration and atherosclerotic remodeling (41). PDIA2 expression was upregulated in cancer cells in response to ER stress (42). In our chronic bowel inflammation to the CRC animal model, PDIA2, standing out of other PDIs, significantly upregulated from a chronic inflammation status to cancer development. In addition, PDIA2 overexpression is positively related to primary tumor grading. These findings are complementary to the functions of PDIA2 in colon cancer development.

An interesting finding of this study is the translocation of overexpressed PDIA2 into mitochondria, where the PDIA2 communicates with complexes I, II, IV, and V of the electron transport chain (ETC) that functions as the transport of electrons from the reduced forms of nicotinamide adenine dinucleotide (NADH) or flavin adenine dinucleotide (FADH2) to ubiquinone and protons across the inner membrane of mitochondria and generates a proton gradient to drive ATP production. Among the complexes, complex I, the first and largest protein component of electron transport chain, is particularly often affected by different regulators, and its dysfunction has often been discussed as a mechanism of cancer development (43–45). Although all complexes can be targeted during energetic modification in order to meet the energy demand of physiological or pathological conditions, three of 6 PDIA2-networked mitochondrial proteins are the core subunits of complex I. As a consequence, the interaction of PDIA2 and complexes of ETC represses oxygen consumption and ATP production through oxophosphorylation coupling, resulting in cellular energetic switches from mitochondrial respiration to cytosolic glycolysis.

Glycolysis, also known as the Warburg effect if it occurs under normoxia conditions, is a common signature of both inflammation and cancer (5, 46) and is one of the two energetic pathways in addition to mitochondrial respiration; between them, cells oscillate freely to meet the energy demand of physiological and pathophysiological activities. Acute inflammation temporarily mutes mitochondrial respiration, resulting in compensatory increases in glycolysis to support stress-mediated anabolism; the flexibility of metabolic oscillation is then restored upon the elimination of the stress stimuli (47, 48). In contrast, chronic inflammation, like aging, causes mitochondrial defects and sustains compensatory hyperglycolysis that promotes cell transformation (49, 50). Although the Warburg effect can be conducted by many other factors such as hypoxia-inducible factor-1 (HIF-1) overexpression, oncogene activation, the loss of function of tumor suppressors, activated or deactivated signaling pathways, and the components of the tumor microenvironment, the hyperglycolysis compensatory to mitochondrial defects is an essential part of the “selfish” metabolic reprogramming in cancer development, as glycolysis and its associated branch metabolic pathways perfectly support cell proliferation not only by producing energy but also by providing building materials like pentose, amino acids, one carbon unit, and anti-oxidant products. Thus, the energetic switch from mitochondrial respiration to glycolysis becomes a critical checkpoint where the chronic inflammation accumulates to trigger a cascade response of cancer development (51). To favor metabolic switch by inflammation, mitochondrial energetics can be subdued by targeting mitochondrial membrane proteins, Kreb’s cycle, and the inhibition of the respiratory complexes of the electron transport chain. Our study revealed the overexpression of PDIA2 in response to the chronic stress of colon mucosa linked to the inhibition of respiratory complexes. The dysfunction of ETC complexes by PDIA2 overexpression minimized the mitochondrial oxygen consumption rate and increased the glycolytic capacity; the knockdown expression of PDIA2 reversed the imbalance of mitochondrial respiration and glycolysis. These observations suggested a key role of ER stress–mitochondrial respiration axis in promoting inflammation to cancer transformation, and PDIA2 acts as a molecular link. Our findings promised a clue to targeted cancer therapies.

## Conclusion

This study highlighted the role of ER stress−mitochondrial respiration axis in prompting chronic colitis to colon cancer transformation. The inflammatory ER stress promotes metabolic modification, and PDIA2 serves as a molecule link between ER stress and energetic reprogramming. Our study also suggested PDIA2 as a potential diagnostic biomarker of colon cancer. However, we deem that PDIA2, even though important, is not the only factor of ER stress in promoting a cancerous transformation of inflammation; this is evidenced by the fact that the repression of PDIA2 only abates but does not prevent tumor development from inflammatory ER stress. In addition, several questions need to be clarified in future to linearize the underlying mechanisms of PDIA2 actions: 1: what is the underlying mechanism of PDIA2 translocation to mitochondria? 2: Why does PDIA2 overwhelm other PDI family members in inducing a mitochondrial defect and prompting colitis to colon cancer transformation? 3: How does the chronic ER stress signaling appropriately modify mitochondrial respiration but eschew apoptosis to develop cancer? 4: Do other ER stress molecules participate in the malignant transformation of chronic colitis? 5: Does the PDIA2-asociated mechanism in the malignant transformation of chronic colitis apply to other types of inflammation to cancerous transformation? Answering these questions should help to prevent chronic inflammation to cancer transformation and design molecularly targeted cancer therapies.

## Data Availability Statement

The original contributions presented in the study are included in the article/**Supplementary Material**. Further inquiries can be directed to the corresponding authors.

## Ethics Statement

The studies involving human participants were reviewed and approved by the Ethics Committee of the Shanghai Public Health Clinical Center (No. 2019-S035-02). The patients/participants provided their written informed consent to participate in this study. The animal study was reviewed and approved by the Ethics Committee of Shanghai Public Health Clinic Center (2014-A032-02).

## Author Contributions

JT, LY, AW, and JLZ contributed equally to this work. TL and LJZ conceived ideas, designed the experiments, and wrote the manuscript. JT, LY, JPZ, AW, HS, JLZ, LN, LX, YF, and XZ performed the experiments and analyzed the results. SYL, LYZ, and XM analyzed the data. SXL and LL enrolled patients and collected tissue samples. All authors contributed to the article and approved the submitted version.

## Funding

This study was financially supports by grants of the National Natural Science Funds (32070946) and the National Basic Research Program of China (973 Program) (2011CB910702).

## Conflict of Interest

The authors declare that the research was conducted in the absence of any commercial or financial relationships that could be construed as a potential conflict of interest.

## Publisher’s Note

All claims expressed in this article are solely those of the authors and do not necessarily represent those of their affiliated organizations, or those of the publisher, the editors and the reviewers. Any product that may be evaluated in this article, or claim that may be made by its manufacturer, is not guaranteed or endorsed by the publisher.

## Supplementary Material

Supplementary Figure S1 Body weight changes of mice after saline or AOM/DSS treatment.

Supplementary Figure S2 Verification of the exclusion of muscle and film layers from stripped colon mucosa by histology exam. **(A)** Normal colon mucosa. **(B)** Colon mucosa after AOM/DSS treatment.

Supplementary Figure S3 Protein expression of ER stress and electron transport chain (ETC) complex. **(A)** expression of ER stress-associated proteins. **(B)** expression of ETC complexes.

Supplementary Figure S4 Cell apoptosis detection by flow cytometry. Wild type HT29 cells, control knockdown C0 cells, and PDIA2 gene specific knockdown C3 cells were co-stained with annexin-FITC and propidium iodide (PI), followed by flow cytometry analysis using Flow cytometer (Beckman, USA).

Supplementary Figure S5 Subcutaneous tumor generated from the incomplete PDIA2 knockdown cells restored glycolysis. Single cell suspensions were prepared from subcutaneous tumors and were assessed for metabolic changes using mitochondrial stress and glycolysis stress kits. **(A)** mitochondrial stress assay of subcutaneous tumor cells generated from wild-type and PDIA2 knockdown cells. FCCP: Carbonyl cyanide-4 (trifluoromethoxy) phenylhydrazone. **(B)** Comparison of basal mitochondrial respiration (oxygen consumption rate, OCR) and respiratory reserve between wilt-type and PDIA2 knockdown cell-generated tumor cells. **(C)** Comparison of proton leak (oxophosphorylation uncoupling) and ATP production between wilt-type and PDIA2 knockdown cell-generated tumor cells. **(D)** Glycolysis stress assay of wild-type and PDIA2 knockdown cell-generated tumor cells. 2-DG: 2-Deoxy-D-glucose. **(E)** Comparison of basal glycolysis (extracellular acidification rate, ECAR), glycolytic capacity and reserve between wild-type and PDIA2 knockdown cell-generated subcutaneous tumor cells.

Supplementary Figure S6 Microarray analysis of PDIA2 expression in paired human colon cancer and adjacent tissues. **(A)** The tissue microarray of 90 pairs of colon cancer and their adjacent colon tissues, of which 16 pairs were removed from quantification of PDIA2 expression due to tissue drop-off. **(B)** Magnification of a representative microarray data. T: cancer tissue, A: adjacent tissue.

Supplementary Figure S7 The expression of PDI isoforms in human colon cancer and cancer adjacent tissues detected by real-time qPCR technology.
