# Supplementary figures and images for: PDIA2 Bridges Endoplasmic Reticulum Stress and Metabolic Reprogramming During Malignant Transformation of Chronic Colitis
Source: Front Oncol. 2022 Jul 4;12:836087. doi: 10.3389/fonc.2022.836087 (PMC9289542; doi:10.3389/fonc.2022.836087)

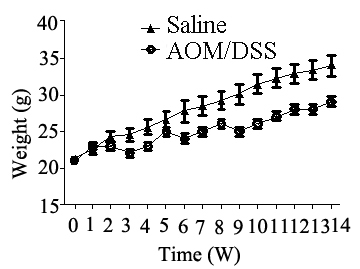

Supplement: Supplementary Figure S1 — Body weight changes of mice after saline or AOM/DSS treatment. [file Image_1.jpeg]

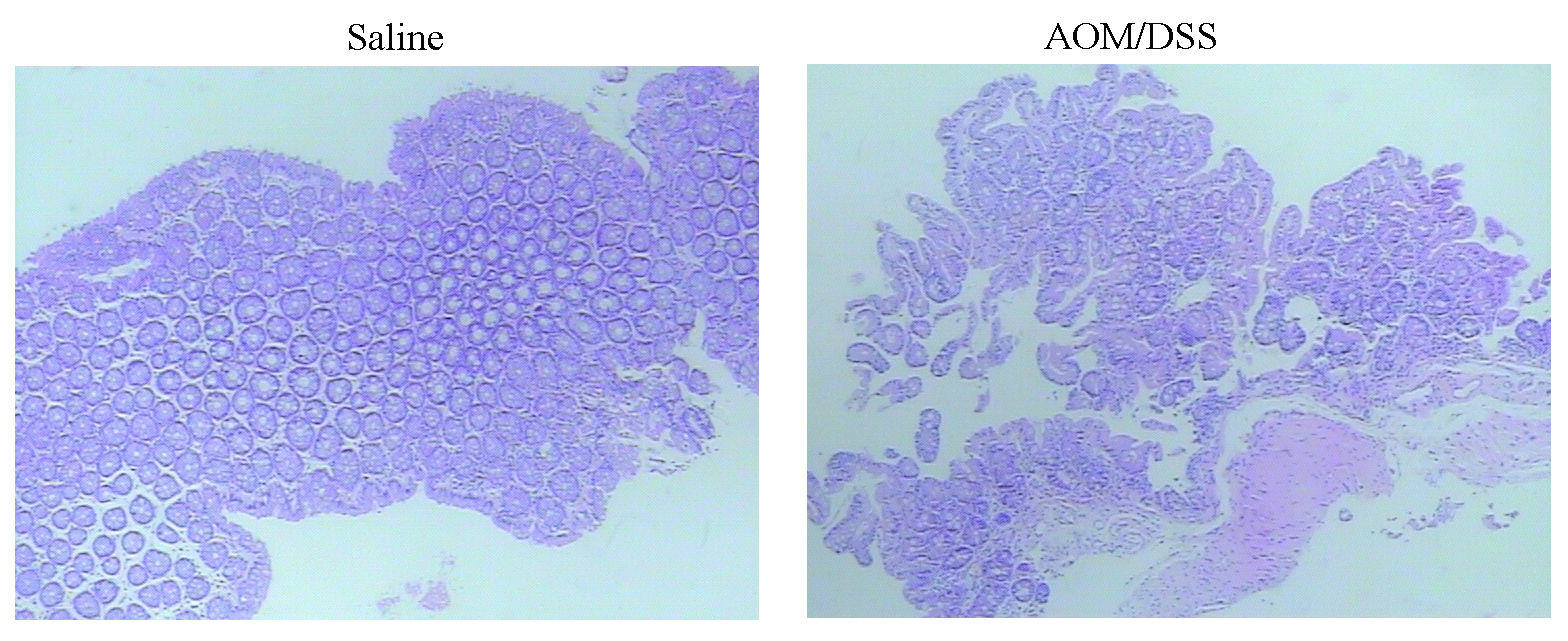

Supplement: Supplementary Figure S2 — Verification of the exclusion of muscle and film layers from stripped colon mucosa by histology exam. (A) Normal colon mucosa. (B) Colon mucosa after AOM/DSS treatment. [file Image_2.tif]

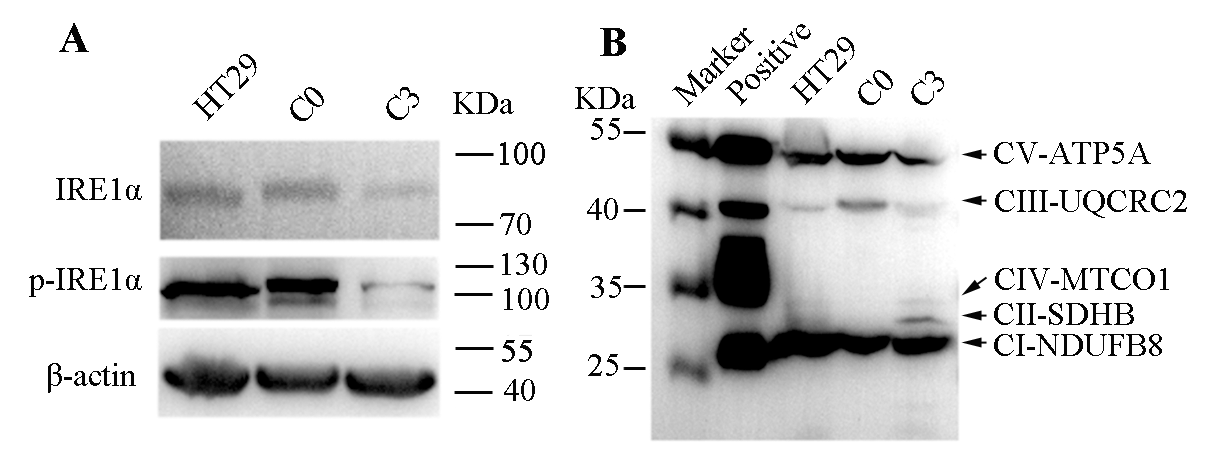

Supplement: Supplementary Figure S3 — Protein expression of ER stress and electron transport chain (ETC) complex. (A) expression of ER stress-associated proteins. (B) expression of ETC complexes. [file Image_3.tif]

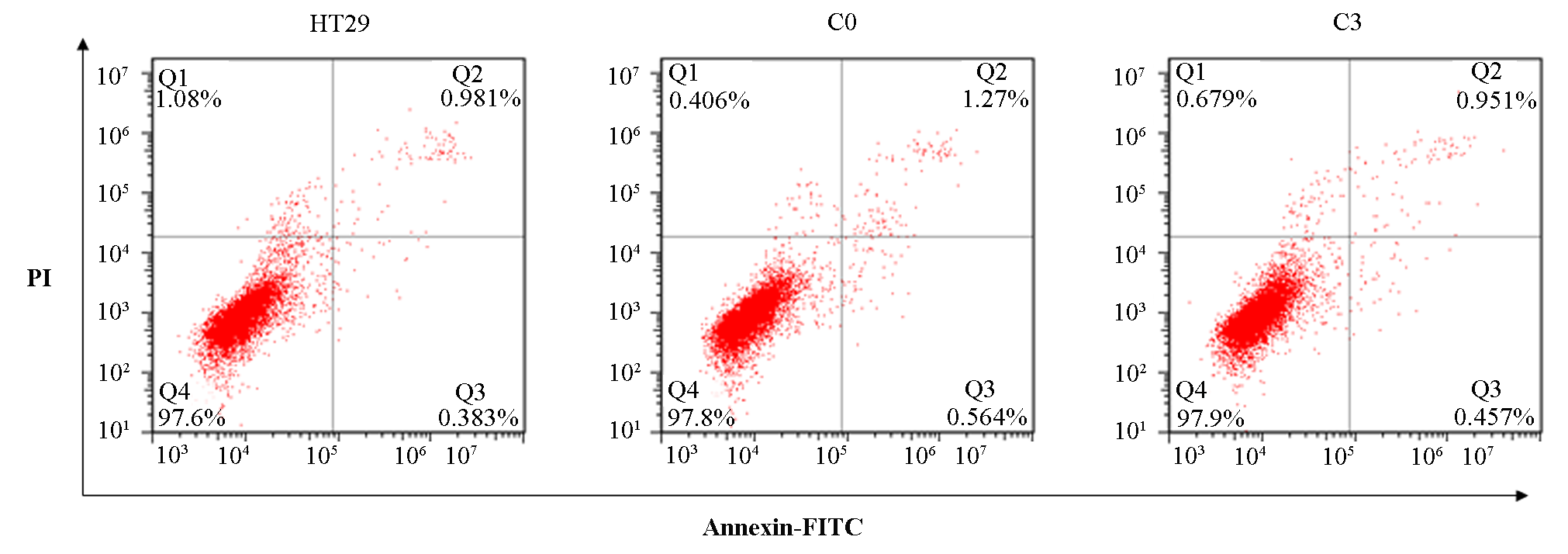

Supplement: Supplementary Figure S4 — Cell apoptosis detection by flow cytometry. Wild type HT29 cells, control knockdown C0 cells, and PDIA2 gene specific knockdown C3 cells were co-stained with annexin-FITC and propidium iodide (PI), followed by flow cytometry analysis using Flow cytometer (Beckman, USA). [file Image_4.tif]

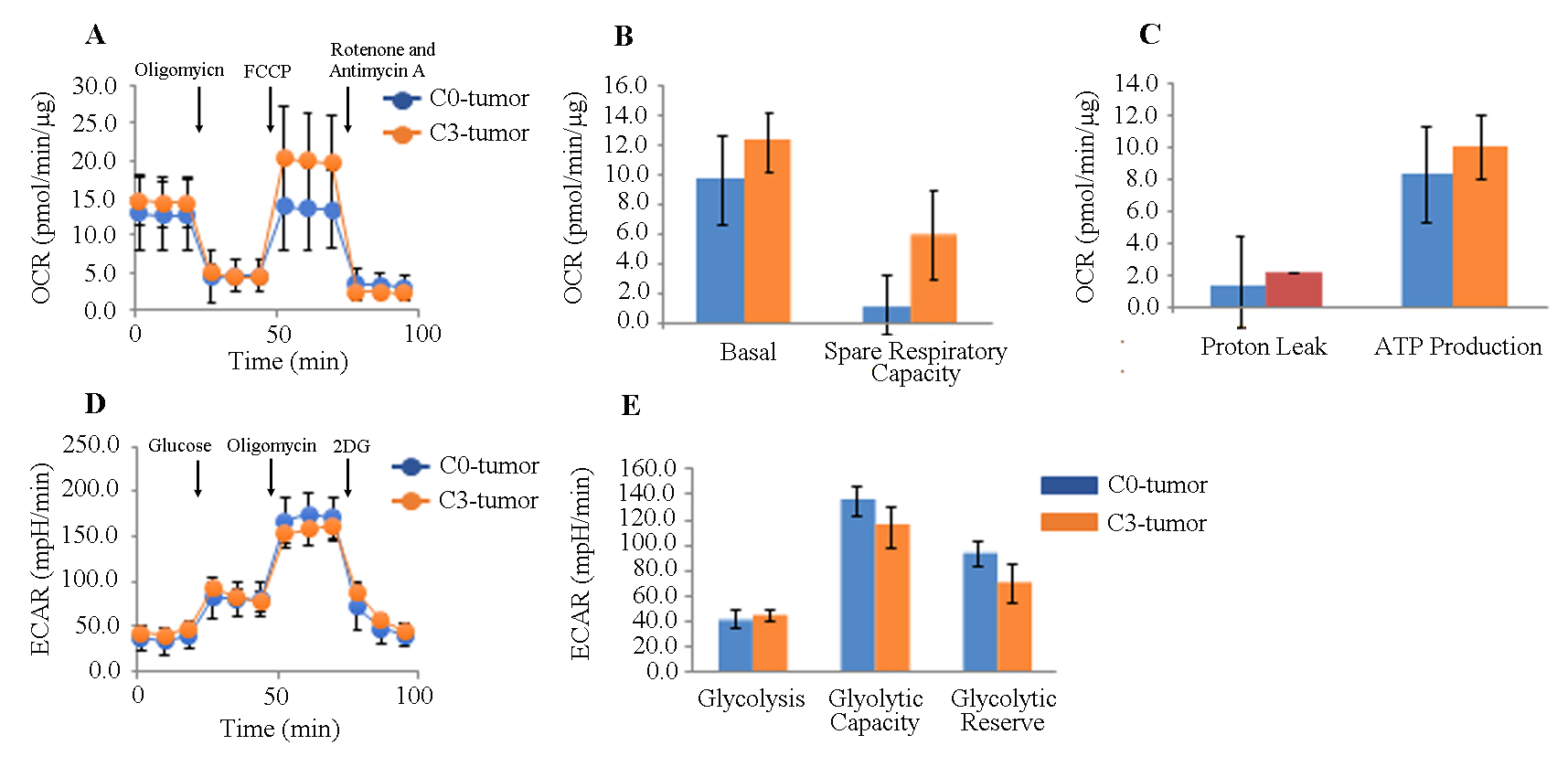

Supplement: Supplementary Figure S5 — Subcutaneous tumor generated from the incomplete PDIA2 knockdown cells restored glycolysis. Single cell suspensions were prepared from subcutaneous tumors and were assessed for metabolic changes using mitochondrial stress and glycolysis stress kits. (A) mitochondrial stress assay of subcutaneous tumor cells generated from wild-type and PDIA2 knockdown cells. FCCP: Carbonyl cyanide-4 (trifluoromethoxy) phenylhydrazone. (B) Comparison of basal mitochondrial respiration (oxygen consumption rate, OCR) and respiratory reserve between wilt-type and PDIA2 knockdown cell-generated tumor cells. (C) Comparison of proton leak (oxophosphorylation uncoupling) and ATP production between wilt-type and PDIA2 knockdown cell-generated tumor cells. (D) Glycolysis stress assay of wild-type and PDIA2 knockdown cell-generated tumor cells. 2-DG: 2-Deoxy-D-glucose. (E) Comparison of basal glycolysis (extracellular acidification rate, ECAR), glycolytic capacity and reserve between wild-type and PDIA2 knockdown cell-generated subcutaneous tumor cells. [file Image_5.jpeg]

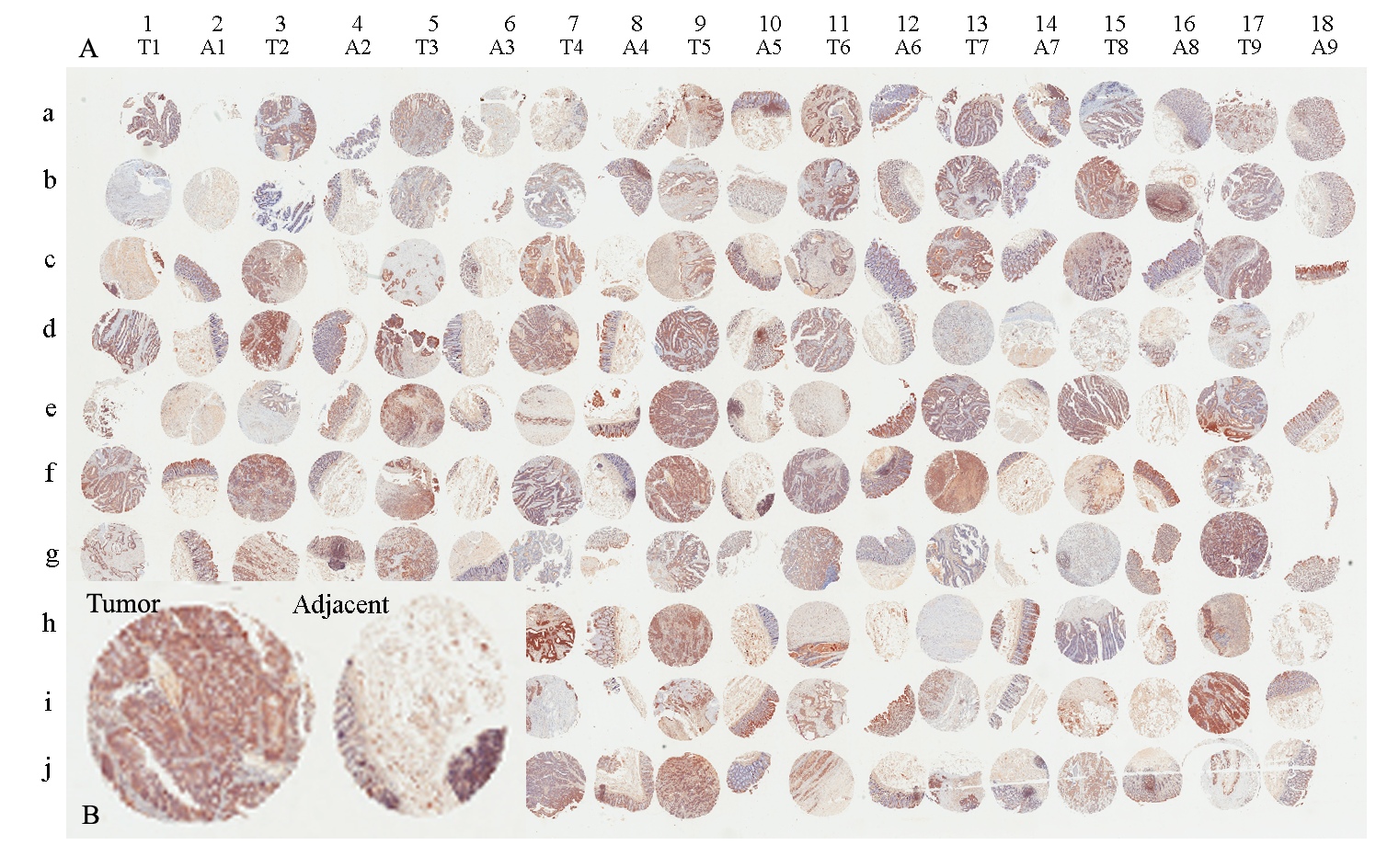

Supplement: Supplementary Figure S6 — Microarray analysis of PDIA2 expression in paired human colon cancer and adjacent tissues. (A) The tissue microarray of 90 pairs of colon cancer and their adjacent colon tissues, of which 16 pairs were removed from quantification of PDIA2 expression due to tissue drop-off. (B) Magnification of a representative microarray data. T: cancer tissue, A: adjacent tissue. [file Image_6.tif]

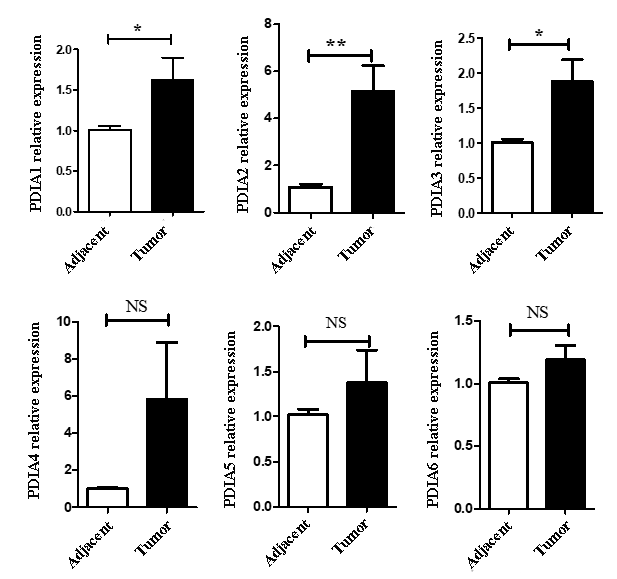

Supplement: Supplementary Figure S7 — The expression of PDI isoforms in human colon cancer and cancer adjacent tissues detected by real-time qPCR technology. [file Image_7.tif]
