# Supplementary material for: PDIA2 Bridges Endoplasmic Reticulum Stress and Metabolic Reprogramming During Malignant Transformation of Chronic Colitis
Source: Front Oncol. 2022 Jul 4;12:836087. doi: 10.3389/fonc.2022.836087 (PMC9289542; doi:10.3389/fonc.2022.836087)
Supplement: Supplementary file 9 [file Table_2.docx]

**Table S2|** shRNA sequences and their targeted sequences on PDIA2

| **Targeted ID** | **Targeted sequence** |
| --- | --- |
| PDIA2-shRNA (C1) | CCGCGGCTCTTTCAGCAGTTT |
| PDIA2-shRNA (C2) | GAGCACGTGCTGCAGTACTTT |
| PDIA2-shRNA (C3) | GCCCTGCTGGTGGAATTCTAT |
| **ShRNA ID** | **5’-stem-loop-stem-3’** |
| PDIA2-shRNA(C1)-a | **Ccgg**CCGCGGCTCTTTCAGCAGTTT**CTCG**AGAAACTGCTGAAAGAGCCGCGG**TTTTTg** |
| PDIA2-shRNA(C1)-b | **aattcaaaaa**CCGCGGCTCTTTCAGCAGTTT**CTCGAG**AAACTGCTGAAAGAGCCGCGG |
| PDIA2-shRNA(C2)-a | **Ccgg**GAGCACGTGCTGCAGTACTTT**CTCGAG**AAAGTACTGCAGCACGTGCTC**TTTTTg** |
| PDIA2-shRNA(C2)-b | **aattcaaaaa**GAGCACGTGCTGCAGTACTTT**CTCGAG**AAAGTACTGCAGCACGTGCTC |
| PDIA2-shRNA(C3)-a | **Ccgg**GCCCTGCTGGTGGAATTCTAT**CTCGAG**ATAGAATTCCACCAGCAGGGC**TTTTTg** |
| PDIA2-shRNA(C3)-b | **aattcaaaaa**GCCCTGCTGGTGGAATTCTAT**CTCGAG**ATAGAATTCCACCAGCAGGGC |
