# Supplementary material for: PDIA2 Bridges Endoplasmic Reticulum Stress and Metabolic Reprogramming During Malignant Transformation of Chronic Colitis
Source: Front Oncol. 2022 Jul 4;12:836087. doi: 10.3389/fonc.2022.836087 (PMC9289542; doi:10.3389/fonc.2022.836087)
Supplement: Supplementary file 10 [file Table_3.docx]

**Table S3|** The primers of PDI isoforms.

Six pairs of clinical colon cancer and their adjacent colon tissues were used.

| Name | Characters | Primers |
| --- | --- | --- |
| PDIA1 | forward | CTCGACAAAGATGGGGTTGT |
|  | reverse | GCAAGAACAGCAGGATGTGA |
| PDIA2 | forward | GGAGTTTGGTGTGACGGAGT |
|  | reverse | AGGTCCTGGAAGAAGCCAAT |
| PDIA3 | forward | AAGCTCAGCAAAGACCCAAA |
|  | reverse | CACTTAATTCACGGCCACCT |
| PDIA4 | forward | CATCAAGGACTTCGTGCTGA |
|  | reverse | TTCACCTCCCCAGCATAGTC |
| PDIA5 | forward | AGTGGATGCAAAACCCTGAG |
|  | reverse | CATCTTTGAAGGCATCAGCA |
| PDIA6 | forward | TTCTGGCAGTGAATGGTCTG |
|  | reverse | GCTGCTTTCTTCCATTCTGG |
| GAPDH | forward | GAAGGTGAAGGTCGGAGTC |
|  | reverse | GAAGATGGTGATGGGATTTC |
