# Supplementary material for: PDIA2 Bridges Endoplasmic Reticulum Stress and Metabolic Reprogramming During Malignant Transformation of Chronic Colitis
Source: Front Oncol. 2022 Jul 4;12:836087. doi: 10.3389/fonc.2022.836087 (PMC9289542; doi:10.3389/fonc.2022.836087)
Supplement: Supplementary file 12 [file Table_5.docx]

**TABLE S5**| The colon tumor sizes in AOM/DSS treated mice.

| Mouse ID | | | Tumor size (mm^2^) | | | | | | |
| --- | --- | --- | --- | --- | --- | --- | --- | --- | --- |
|  |  | Tumor 1 | Tumor 2 | Tumor 3 | Tumor 4 | Tumor 5 | Tumor 6 | Tumor 7 | Tumor 8 |
| 7 weeks | 1 | 6.61 |  |  |  |  |  |  |  |
|  | 2 |  |  |  |  |  |  |  |  |
|  | 3 | 4.41 |  |  |  |  |  |  |  |
|  | 4 | 7.92 |  |  |  |  |  |  |  |
|  | 5 | 8.64 |  |  |  |  |  |  |  |
|  | 6 |  |  |  |  |  |  |  |  |
|  | 7 |  |  |  |  |  |  |  |  |
|  | 8 | 7.35 |  |  |  |  |  |  |  |
|  | 9 |  |  |  |  |  |  |  |  |
|  | 10 |  |  |  |  |  |  |  |  |
| Average (±STD) | | 6.99±1.62 | | | | | | | |
| 14 weeks | 1 | 8.24 | 6.53 |  |  |  |  |  |  |
|  | 2 | 8.09 | 13.84 | 5.87 | 7.18 | 10.11 |  |  |  |
|  | 3 | 8.06 | 22.07 | 5.29 |  |  |  |  |  |
|  | 4 | 11.70 | 10.76 | 13.95 | 8.73 | 4.88 | 4.20 |  |  |
|  | 5 | 4.77 | 8.17 | 13.72 | 9.02 | 7.10 | 3.66 | 14.53 | 6.70 |
|  | 6 | 9.34 | 10.37 |  |  |  |  |  |  |
|  | 7 | 11.76 | 12.11 | 12.06 | 11.29 | 10.21 | 9.64 | 16.81 | 9.36 |
|  | 8 | 15.36 | 5.97 | 4.23 | 7.87 |  |  |  |  |
|  | 9 | 5.21 | 4.00 |  |  |  |  |  |  |
|  | 10 | 6.27 | 9.89 |  |  |  |  |  |  |
| Average (±STD) | | 9.35±3.87 | | | | | | | |
