# Supplementary material for: PDIA2 Bridges Endoplasmic Reticulum Stress and Metabolic Reprogramming During Malignant Transformation of Chronic Colitis
Source: Front Oncol. 2022 Jul 4;12:836087. doi: 10.3389/fonc.2022.836087 (PMC9289542; doi:10.3389/fonc.2022.836087)
Supplement: Supplementary file 15 [file Table_8.docx]

**TABLE S8|** PDIA2 interacting proteins in metabolic and endoplasmic reticulum processing pathways.

| **Metabolic pathways** |
| --- |
| CDIPT,GAPDHS,OGDH,ATP6V1B1,DAD1,PGLS,ATP6V1E1,PI4KA,GYS2,ALDH2,PIK3C3,LPCAT2,SPTLC1,CPOX,PPAT,MCCC1,SQLE,PIK3C2A,CERS2,ACAD8,ATP5O,HK2,MTMR14,ADH5,LPCAT4,GYS1,NDUFV1,ALDH3A2,GPAA1,AGK,FDPS,MT-CO2,MT-ND2,FH,PLA2G4A,GCLM,PGK1,SDHB,SEPHS1,GMDS,IDI1,SACM1L,GALNT3,DAK,ALG3,TST,NDUFS1,EARS2,EBP,UGT1A3,ACSL1,CEPT1,DGAT1 |
| **Protein processing in endoplasmic reticulum** |
| SSR1,CANX,DAD1,LMAN1,DNAJB1,HSPBP1,DERL1,HSPA4L,DNAJA2,P4HB,TXNDC5,SEC13,SAR1B,DNAJC3 |
